# Supplementary material for: Genotoxicity Evaluation of Propyl-Propane-Thiosulfinate (PTS) from Allium genus Essential Oils by a Combination of Micronucleus and Comet Assays in Rats
Source: Foods. 2021 May 1;10(5):989. doi: 10.3390/foods10050989 (PMC8147216; doi:10.3390/foods10050989)
Supplement: Supplementary file 1 [file foods-10-00989-s001.zip › foods-1179045-supplementary.pdf]

Table S1: measures of the% DNA in tail for the liver of the standard comet assay and the modified comet assay with enzymes in their different treatments: buffer F, FPG, Edo III.

Groups	Sex	N	Doses	SD break (%DNA in tail)	Buffer F (%DNA in tail)	FPG (%DNA in tail)	ENDOIII (%DNA in tail)
Negative Control	♂	1	-	1,42	0,94	2,18	2,71
	♂	2	-	0,13	0,12	0,39	0,15
	♂	3	-	0,12	0,09	0,18	1,59
	♂	4	-	0,04	0,10	0,58	0,36
	♂	5	-	0,05	0,18	0,95	0,67
	♀	1	-	0,15	0,01	0,02	0,03
	♀	2	-	0,23	0,02	0,16	0,13
	♀	3	-	0,33	0,02	0,05	1,05
	♀	4	-	0,40	0,23	1,28	0,43
	♀	5	-	0,05	0,03	0,07	0,13
Solvent Control	♂	1	-	0,15	1,36	1,48	2,26
	♂	2	-	0,18	0,98	1,06	1,34
	♂	3	-	0,04	0,08	1,31	0,62
	♂	4	-	0,03	0,06	0,13	0,09
	♂	5	-	0,04	0,01	0,09	0,01
	♀	1	-	0,36	0,01	0,02	0,03
	♀	2	-	0,05	0,01	0,02	0,01
	♀	3	-	0,10	0,01	0,29	0,17

	♀	4	-	0,02	0,01	0,09	0,32
	♀	5	-	0,02	0,06	0,06	0,24
Positive Control (EMS[1])	♂	1	200.0mg/Kg	34,32	36,59	48,13	51,36
	♂	2		33,75	39,48	53,62	45,16
	♂	3		30,00	28,37	37,48	54,83
	♀	1		24,23	25,32	30,17	36,32
	♀	2		23,55	18,40	23,22	28,18
	♀	3		19,36	17,12	25,30	30,83
0,02P0,07TS	♂	1	5.5 mg/Kg	0,21	0,04	2,40	0,13
	♂	2		0,02	0,01	0,02	0,12
	♂	3		0,01	0,03	0,06	0,51
	♂	4		0,02	0,02	0,39	0,50
	♂	5		0,03	0,02	0,21	0,04
	♀	1		0,01	0,01	0,05	0,04
	♀	2		0,01	0,01	0,01	0,06
	♀	3		0,16	0,02	0,07	0,04
	♀	4		0,04	0,02	0,03	0,06
	♀	5		0,08	0,03	0,14	1,29
	♂	1	17.4 mg/Kg	0,02	0,01	0,02	0,22
	♂	2		0,01	0,16	0,26	0,31
	♂	3		0,05	0,07	0,37	0,37
	♂	4		0,06	0,05	0,08	0,74

	♂	5		0,03	0,01	0,02	0,03
	♀	1		0,07	0,02	0,38	0,23
	♀	2		0,07	0,01	0,04	0,10
	♀	3		0,39	0,00	0,02	0,12
	♀	4		0,31	0,00	0,88	0,22
	♀	5		0,01	0,04	0,09	0,84
	♂	1	55.0 mg/Kg	0,10	0,00	0,02	0,01
	♂	2		0,13	0,01	0,09	0,03
	♂	3		0,05	0,09	0,44	0,25
	♂	4		0,03	0,18	1,41	0,61
	♂	5		0,42	0,04	0,12	0,23
	♀	1		0,05	0,00	0,04	0,40
	♀	2		0,01	0,01	0,93	0,30
	♀	3		0,01	0,00	0,01	1,17
	♀	4		0,01	0,01	0,10	0,03
	♀	5		0,06	0,00	0,00	1,08

The values are expressed as individual measurements

Table S2: measures of the% DNA in tail for the stomach of the standard comet assay and the modified comet assay with enzymes in their different treatments: buffer F, FPG, Edo III.

Groups	Sex	N	Doses	SD break (%DNA in tail)	Buffer F (%DNA in tail)	FPG (%DNA in tail)	ENDOIII (%DNA in tail)
Negative Control	♂	1	-	0,01	2,08	2,64	4,54
	♂	2	-	0,02	0,13	0,80	0,60
	♂	3	-	0,00	1,02	1,08	6,01
	♂	4	-	0,01	1,20	2,80	2,53
	♂	5	-	0,00	1,08	2,11	4,17
	♀	1	-	0,04	3,45	5,19	8,27
	♀	2	-	0,03	0,51	1,40	1,72
	♀	3	-	0,04	0,41	1,57	2,86
	♀	4	-	0,05	0,32	1,22	4,25
	♀	5	-	0,05	0,24	1,03	0,25
Solvent Control	♂	1	-	0,03	0,75	2,05	2,05
	♂	2	-	0,10	0,42	1,75	1,65
	♂	3	-	0,05	0,38	0,99	0,55
	♂	4	-	0,02	0,65	2,54	2,67
	♂	5	-	0,00	1,19	2,65	2,02
	♀	1	-	0,02	0,28	0,47	0,85
	♀	2	-	0,05	0,44	1,14	0,77
	♀	3	-	0,05	0,09	0,27	0,28

	♀	4	-	0,06	1,10	2,09	1,16
	♀	5	-	0,03	0,20	0,22	0,22
Positive Control (EMS[1])	♂	1	200.0mg/Kg	29,20	34,81	49,81	46,21
	♂	2		12,72	52,29	64,42	67,13
	♂	3		20,56	59,19	84,17	83,28
	♀	1		15,03	43,95	60,97	54,78
	♀	2		16,73	39,90	54,76	64,55
	♀	3		12,49	45,87	55,87	61,71
PTS	♂	1	5.5 mg/Kg	0,00	0,16	0,27	0,18
	♂	2		0,10	0,18	0,19	0,59
	♂	3		0,00	0,42	0,93	1,93
	♂	4		0,01	0,35	0,58	2,65
	♂	5		0,01	0,27	0,98	0,41
	♀	1		0,00	0,22	0,65	0,23
	♀	2		0,00	0,17	0,28	0,29
	♀	3		0,00	0,52	2,67	1,78
	♀	4		0,00	0,04	0,09	0,30
	♀	5		0,00	0,03	0,08	0,13
	♂	1	17.4 mg/Kg	0,03	0,54	0,59	0,85
	♂	2		0,01	0,01	5,42	0,25
	♂	3		0,00	0,46	1,46	0,65
	♂	4		0,01	1,02	1,49	2,10

	♂	5		0,01	3,70	4,97	7,05
	♀	1		0,00	0,21	0,52	0,85
	♀	2		0,00	0,07	0,46	0,30
	♀	3		0,02	0,20	0,34	0,26
	♀	4		0,00	1,01	2,09	7,02
	♀	5		0,03	0,36	1,28	0,70
	♂	1	55.0 mg/Kg	0,14	0,40	1,53	0,57
	♂	2		0,00	0,17	0,64	0,44
	♂	3		0,05	0,31	1,34	0,94
	♂	4		0,02	0,61	1,41	6,07
	♂	5		0,03	1,71	3,62	3,08
	♀	1		0,00	0,13	2,80	4,53
	♀	2		0,00	2,18	5,93	5,62
	♀	3		0,00	0,77	1,03	2,87
	♀	4		0,00	0,35	0,44	1,04
	♀	5		0,02	0,38	0,48	0,55

The values are expressed as individual measurements.

Table S3: measures of the% DNA in tail for the blood of the standard comet assay and the modified comet assay with enzymes in their different treatments: buffer F, FPG, Edo III.

Groups	Sex	N	Doses	SD break (%DNA in tail)	Buffer F (%DNA in tail)	FPG (%DNA in tail)	ENDOIII (%DNA in tail)
Negative Control	♂	1	-	1,51	0,82	1,10	2,04
	♂	2	-	0,18	0,34	0,91	1,13
	♂	3	-	0,01	0,17	0,51	0,95
	♂	4	-	0,16	0,03	0,15	0,06
	♂	5	-	0,03	0,45	0,75	1,03
	♀	1	-	0,14	0,03	0,23	0,19
	♀	2	-	0,36	0,06	0,36	0,10
	♀	3	-	0,04	0,12	0,14	0,20
	♀	4	-	0,02	0,06	0,42	0,52
	♀	5	-	0,16	0,04	0,28	0,26
Solvent Control	♂	1	-	0,29	0,10	0,19	0,15
	♂	2	-	0,47	0,08	0,27	0,11
	♂	3	-	0,06	0,46	1,33	1,19
	♂	4	-	1,16	0,37	0,67	0,57
	♂	5	-	0,10	0,06	0,21	0,44
	♀	1	-	0,06	0,01	0,07	0,76
	♀	2	-	0,02	0,03	0,15	0,35
	♀	3	-	0,01	0,10	0,18	0,15

	♀	4	-	0,04	0,04	0,93	0,08
	♀	5	-	0,03	0,08	1,18	0,58
Positive Control (EMS[1])	♂	1	200.0mg/Kg	43,70	23,65	33,63	35,45
	♂	2		67,48	21,78	38,23	37,70
	♂	3		56,65	20,34	39,90	40,59
	♀	1		28,64	20,45	41,10	40,99
	♀	2		35,55	24,13	32,03	39,91
	♀	3		41,77	19,54	34,19	34,38
PTS	♂	1	5.5 mg/Kg	0,31	0,03	0,25	0,13
	♂	2		0,03	0,00	0,32	0,08
	♂	3		0,08	0,04	0,19	0,04
	♂	4		0,22	0,02	0,13	0,10
	♂	5		0,18	0,04	0,09	0,24
	♀	1		0,24	0,03	1,35	1,35
	♀	2		0,64	0,04	1,14	0,08
	♀	3		0,05	0,06	0,08	0,41
	♀	4		0,23	0,05	0,55	0,13
	♀	5		0,29	0,07	0,13	0,26
	♂	1	17.4 mg/Kg	0,02	0,03	0,04	0,31
	♂	2		0,63	0,08	0,13	0,24
	♂	3		0,07	0,04	0,31	0,02
	♂	4		0,16	0,02	0,06	0,22

	♂	5		0,08	0,04	0,24	0,33
	♀	1		0,03	0,03	0.05	1,15
	♀	2		0,04	0,04	0.19	0,44
	♀	3		0,02	0,06	0,34	0,09
	♀	4		0,02	0,05	0,05	0,08
	♀	5		0,02	0,07	0,30	0,36
	♂	1	55.0 mg/Kg	0,04	0,04	0,21	2,98
	♂	2		0,01	0,07	0,26	0,23
	♂	3		0,18	0,00	0,00	0,02
	♂	4		0,31	0,07	0,08	0,11
	♂	5		0,04	0,08	0,09	0,17
	♀	1		0,27	0,01	0,18	0,29
	♀	2		0,99	0,11	0,43	0,31
	♀	3		0,31	0,08	0,43	0,25
	♀	4		1,60	0,05	0,81	0,21
	♀	5		0,21	0,01	0,12	0,06

The values are expressed as individual measurements.
